# Uncovering the embryonic development-related proteome and metabolome signatures in breast muscle and intramuscular fat of fast-and slow-growing chickens

**DOI:** 10.1186/s12864-017-4150-3

**Published:** 2017-10-23

**Authors:** Ranran Liu, Hongyang Wang, Jie Liu, Jie Wang, Maiqing Zheng, Xiaodong Tan, Siyuan Xing, Huanxian Cui, Qinghe Li, Guiping Zhao, Jie Wen

**Affiliations:** 1 Institute of Animal Sciences, Chinese Academy of Agricultural Sciences, No. 2 Yuanmingyuan W Rd, Beijing, 100193 People’s Republic of China; 2 State Key Laboratory of Animal Nutrition, Beijing, People’s Republic of China

## Abstract

**Background:**

Skeletal muscle development is closely linked to meat production and its quality. This study is the first to quantify the proteomes and metabolomes of breast muscle in two distinct chicken breeds at embryonic day 12 (ED 12), ED 17, post-hatch D 1 and D 14 using mass spectrometry-based approaches.

**Results:**

Results found that intramuscular fat (IMF) accumulation increased from ED 17 to D 1 and that was exactly the opposite of when most obvious growth of muscle occurred (ED 12 - ED 17 and D 1 - D 14). For slow-growing Beijing-You chickens, Ingenuity Pathway Analysis of 77–99 differential abundance (DA) proteins and 63–72 metabolites, indicated significant enrichment of molecules and pathways related to protein processing and PPAR signaling. For fast-growing Cobb chickens, analysis of 68–95 DA proteins and 56–59 metabolites demonstrated that molecules and pathways related to ATP production were significantly enriched after ED12. For IMF, several rate-limiting enzymes for beta-oxidation of fatty acid (ACADL, ACAD9, HADHA and HADHB) were identified as candidate biomarkers for IMF deposition in both breeds.

**Conclusions:**

This study found that ED 17 - D 1 was the earliest period for IMF accumulation. Pathways related to protein processing and PPAR signaling were enriched to support high capacity of embryonic IMF accumulation in Beijing-You. Pathways related to ATP production were enriched to support the fast muscle growth in Cobb. The beta-oxidation of fatty acid is identified as the key pathway regulating chicken IMF deposition at early stages.

**Electronic supplementary material:**

The online version of this article (10.1186/s12864-017-4150-3) contains supplementary material, which is available to authorized users.

## Background

Chickens are economically significant animals. Skeletal muscle constitutes the largest proportion and most valuable component of meat mass; its development is closely associated with the amount of meat production and its quality. Together with muscular histological traits, such as the type, diameter, density and size of muscle fibers, the deposition of intramuscular fat (IMF) can dramatically promote tenderness of meat and plays an important role in flavor of meat [1–3].

The development of skeletal muscle depends on myogenesis and, to some extent, also on adipogenesis. The muscle mass and IMF content are both determined by cell numbers and unit cell size. Hyperplasia refers to the increasing in cell number or muscle fiber number which occurs mainly in embryonic periods as the numbers of adipocytes and muscle fibers are fixed by the day of hatching [4, 5]. Velleman et al. (2007) observed that breast muscle organizational differences among breeds and between sexes begin to occur between embryonic 20 days (ED 20) and ED 25 in turkeys. Thus, embryonic muscle development has a dramatic impact on the postnatal accretion of muscle mass and its content of IMF [6]. Chartrin et al. (2007) investigated the effects of age (from D 1 to D 98) on IMF of mule ducks and showed that total lipid content in the breast muscle was high at D 1 and indicated that embryonic IMF was necessary for growth and energy requirements at this early stage [7]. Our previous study as reported by Liu et al. (2016) also showed that chicken IMF content was highest at D 1 and the protein expression profile at D 1 differed greatly from other post-hatching ages [8]. The previous molecular studies of muscle development and IMF deposition in poultry have been mainly focused on post-hatchling stages, except for Ouyang’s recent report on the proteomic analysis of embryonic muscle in a local breed [8–10]. Although the importance of embryonic muscle and IMF development was established, underlying molecular mechanisms remain poorly understood.

The Beijing-you (BJY) chicken, called Beijing-fatty chicken, is an indigenous breed in China with good meat quality [11, 12]. When compared with fast-growing commercial broilers, the BJY is favored by local consumers because of the taste, rich fragrance, and tenderness of the meat. The Cobb is a famous genetically improved commercial line with excellent growth rate, and yield, especially of breast muscle. Breast muscle yield could reach more than 20% of live weight after D 28 (www.Cobb-Vantress.com), which is double that (around 10%) of BJY of similar live weight [13].

With the aim of exploring the embryonic development-related proteome and metabolome signatures in breast muscle and its content of IMF, we quantitatively analyzed the proteomes and metabolomes of breast muscle of the two distinct chicken breeds at embryonic day12 (ED 12), ED 17, upon hatching (D 1) and day 14 post-hatching (D 14) using an Isobaric tags for relative and absolute quantitation (iTRAQ) and liquid chromatography/mass spectrometry (LC-MS) based approaches, and verified results from the discovery proteomics by Western blotting of selected proteins. Changing patterns of breast muscle growth and IMF contents at these critical stages of development were also measured.

## Results

### Development of breast muscle and accumulation of intramuscular fat during mid-incubation to early post-hatch growth

The local BJY chickens had distinct breast muscle features when compared with Cobb chickens (the genetically improved broiler line). For the two pre-hatching periods examined, the main muscle growth period appeared to be from ED 12 to ED 17, where the weight increased 1.8 fold in BJY and 8.4 fold in Cobb (Table 1). The fiber density increased marginally from ED 17 to D 1 in Cobb but decreased in BJY (Table 2). That indicates the stronger capacity for hyperplasia in Cobb during the embryonic periods.

**Table 1:** Muscle weights at different stages in Beijing-You and Cobb chickens (g)*

Item	ED 12	ED 17	D 1	D 14
BJY	0.175 ± 0.009^D^	0.310 ± 0.008^C^	0.453 ± 0.022^B^	6.588 ± 0.265^A^
Cobb	0.102 ± 0.006^D^	0.565 ± 0.014^C^	0.730 ± 0.025^B^	26.190 ± 1.087^A^

*Different uppercase superscripts indicate significant differences within the row (*P* < 0.01). On a within-day basis, all differences between two breeds were significant. *n* = 10

**Table 2:** Characteristics of muscle fibers at different stages in Beijing-you and Cobb chickens*

Term	Breeds	Time		
		ED 17	D 1	D 14
Fiber diameter (μm)	BJY	6.85 ± 0.20^C^	7.57 ± 0.14^B^	22.87 ± 0.30^A^
	Cobb	5.10 ± 0.04^B^	4.84 ± 0.07^C^	27.51 ± 0.42^A^
Fiber cross-sectional area (μm^2^)	BJY	28.45 ± 1.42^C^	36.58 ± 1.29^B^	280.79 ± 6.49^A^
	Cobb	16.70 ± 0.24^B^	15.13 ± 0.42^C^	425.79 ± 13.61^A^
Fiber density (fibers/mm^2^)	BJY	29.01 ± 0.40^A^	21.41 ± 0.29^B^	3.04 ± 0.04^C^
	Cobb	47.10 ± 0.80^A^	51.54 ± 3.05^A^	2.54 ± 0.04^B^

*Different uppercase superscripts indicate significant differences (*P* < 0.01) within the row. On a within-day basis, all differences between two breeds were significant.Muscle fibers at ED12 were too small to be reliably measured

**Fig. 1:**
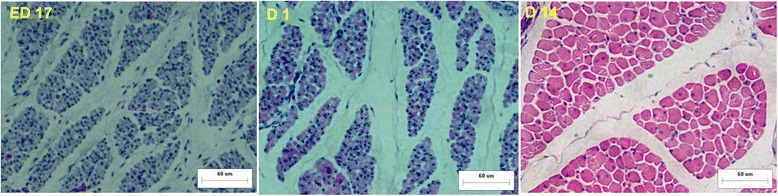
Muscle fibers of the breast muscle stained with hematoxylin and eosin (H&E) in BJY (bar, 60 μm)

**Fig. 2:**
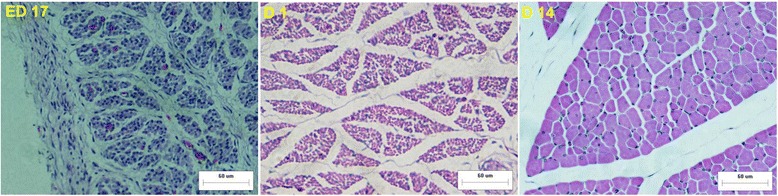
Muscle fibers of the breast muscle stained with hematoxylin and eosin (H&E) in Cobb (bar, 60 μm)

As expected, the muscle growth post-hatching increased much faster than during the pre-hatching periods. The weight increased 14.6 fold in BJY and 35.9 fold in Cobb (Table 1). Correspondingly, the diameters of breast muscle fibers in BJY chickens were markedly smaller and densities of fibers were accordingly greater than those of D 14 Cobb chickens; both variables differed significantly (*P* < 0.01) between the breeds (Table 2, Figs. 1, 2).

**Fig. 3:**
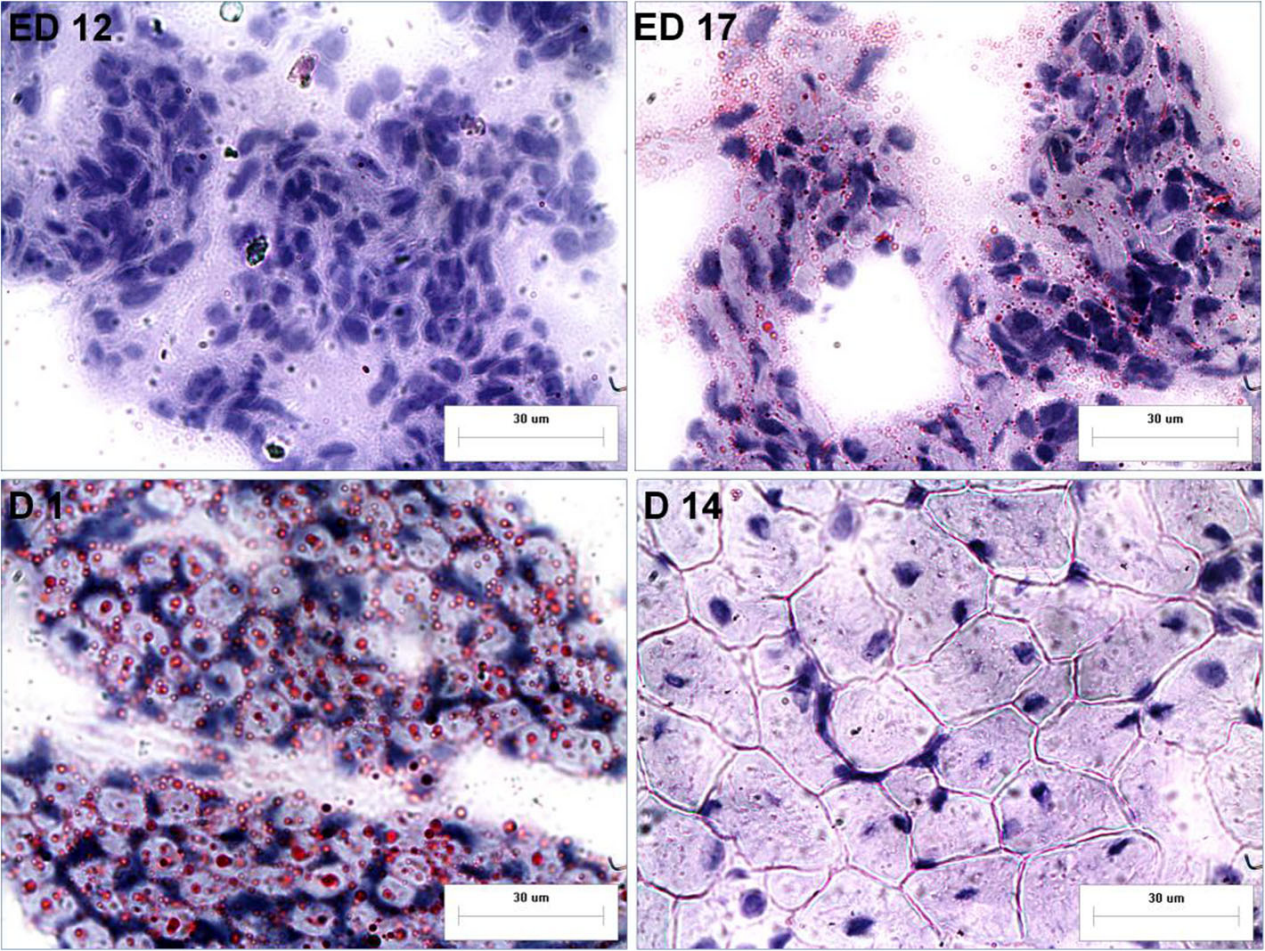
Oil red O staining of breast muscle tissues in BJY at early growth stages (bar, 30 μm)

**Fig. 4:**
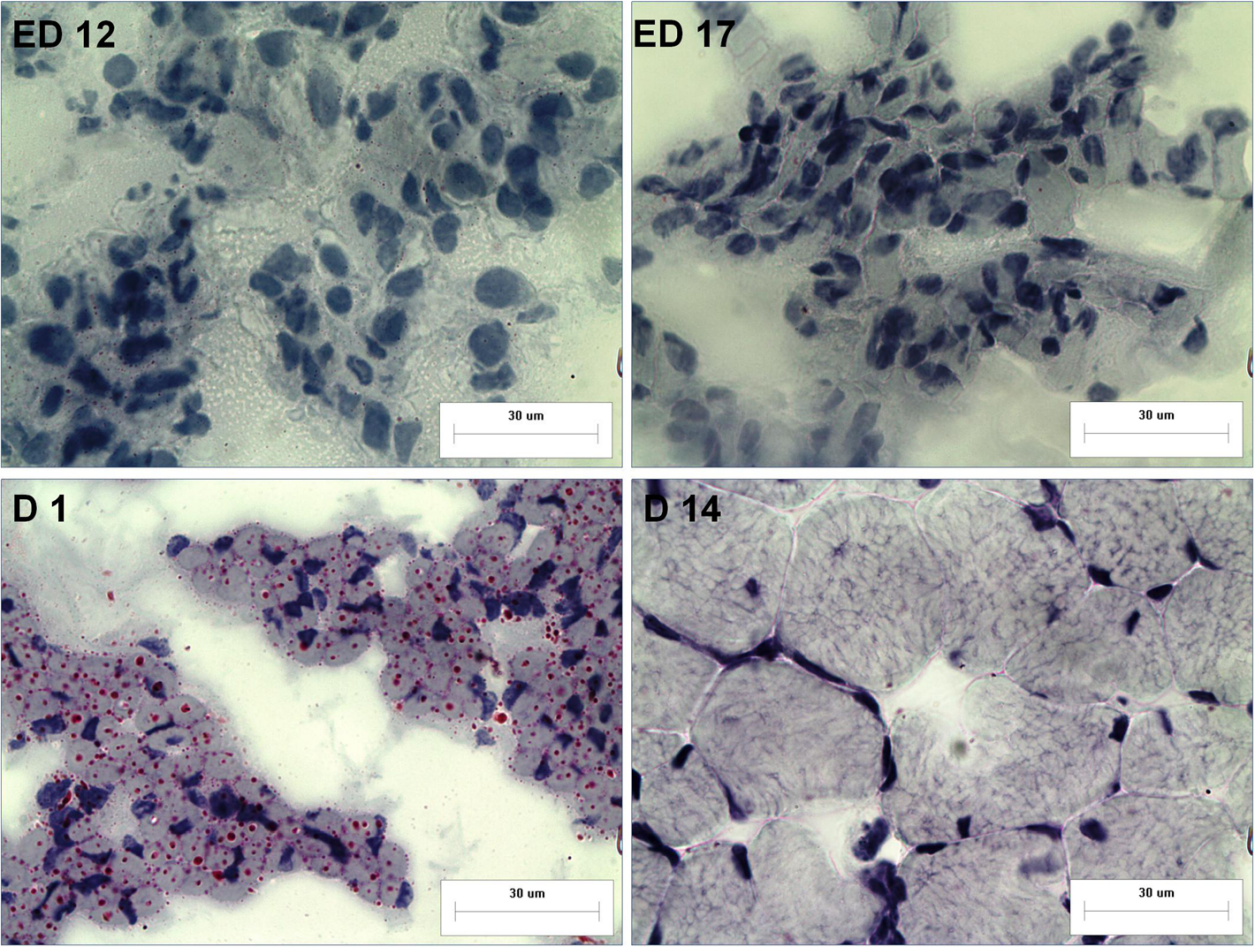
Oil red O staining of breast muscle tissues in Cobb chickens at early growth stages (bar, 30 μm)

The accumulation of IMF in breast muscle at ED 12, ED 17, hatching (D 1) and D 14 was apparent from Oil red O staining (Figs. 3, 4). The maximal content of IMF was observed at D 1 in both BJY and Cobb, indicating that the earliest period of IMF accumulation was from ED 17 to D 1. No detectable fat was observed at D 14 showing that IMF was depleted dramatically between D 1 and D 14. It is worth noting that the major period for IMF accumulation (ED 17 to D 1) was exactly the opposite of when muscle growth occurred (ED 12 – ED 17 and D 1 – D 14).

### Comparative proteomic and metabolomics analysis of breast muscle during mid-incubation to early post-hatch growth

A total of 2502 proteins were identified in the BJYs from 30,958 peptides and 2264 proteins were identified in the Cobbs from 28,456 peptides according to the standard (FDR less than 1%) of protein identification (Additional file 1: Table S1).

Because muscle development proceeds with temporal precision, focus was placed on proteins which differed significantly in abundance between the sequential sampling stages (ED 12 vs ED 17, ED 17 vs D 1 and D 1 vs D 14) in the two breeds. A full list of the quantified proteins and their mean fold-changes of the pairwise comparisons (each with two sub-sample pools) and the corresponding *P*-values is given in Additional file 2: Table S2. According to the criteria stated earlier, a total of 268 and 255 proteins showed significant differential abundance (DA) in BJY and Cobb; they are listed in Table 3.

**Table 3:** The number of differentially abundant proteins and metabolites in Beijing-you and Cobb chickens

Stage Comparison	Beijing-you		Cobb	
	Protein	Metabolite	Protein	Metabolite
ED12-ED17	92	47	95	55
ED17-D1	99	41	92	57
D1-D14	77	52	68	57

**Fig. 5:**
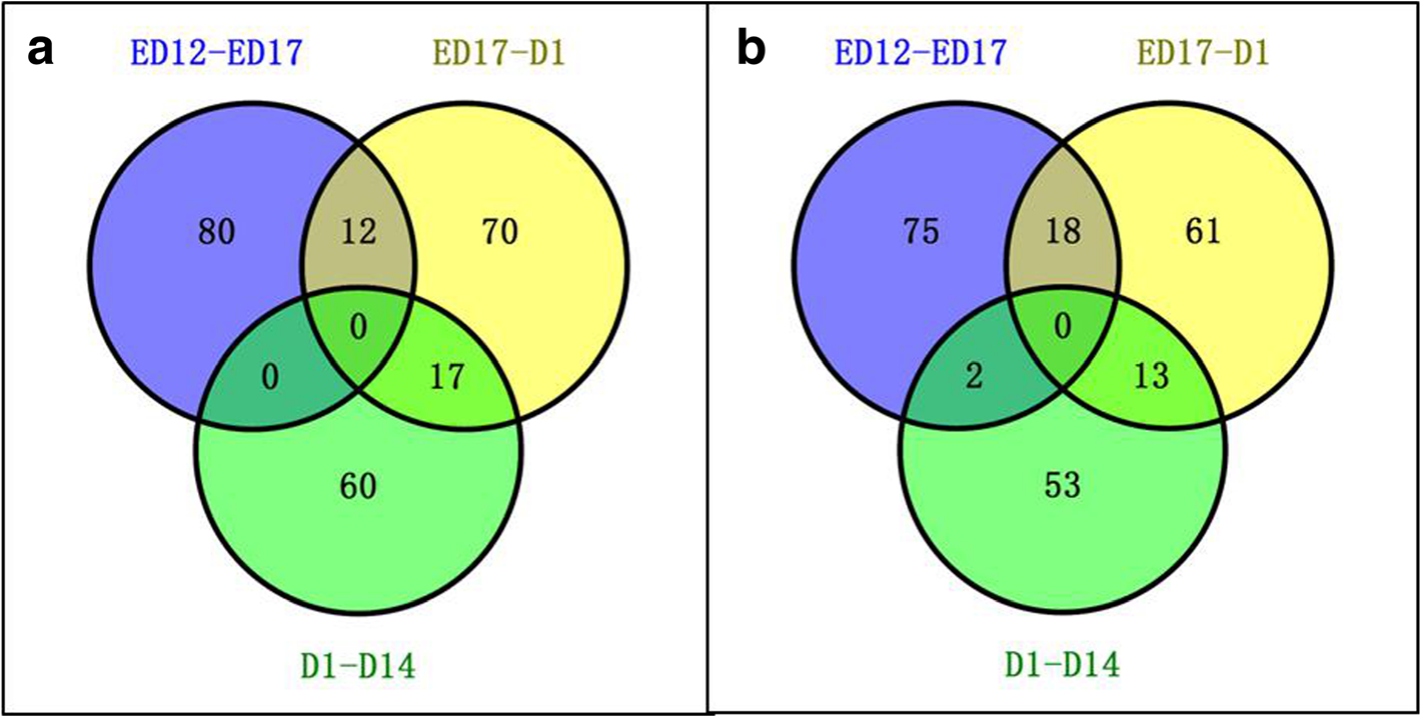
The number of differentially abundant proteins between the sequential sampling stages (ED 12 vs ED 17, ED17 vs D 1 and D 1 vs D 14) in BJY and Cobb chickens

In both breeds, ED 12 to ED 17 and D 1 to D 14 were the main periods for pre- and post-hatching muscle growth (Figs. 1, 2). There was, however, little overlap in differential abundance (DA) proteins between those two stages. For ED 12 to ED 17, the 92 and 95 DA proteins in BJY and Cobb that significantly changed in abundance (Table 2) did not differ between D 1 and D 14, except for two in Cobb). For D 1 to D 14, where striking post-hatching muscle growth occurred, the 77 and 68 DA proteins in BJY and Cobb that significantly changed in abundance (Table 3) differed for the two breeds and did not differ before hatching (Fig. 5).

A total of 41–52 metabolites in the BJYs and 55–57 metabolites in the Cobbs were identified as being differentially abundant according to the discriminating criteria (Table 3 and Additional file 3: Table S3).

### Identification and pathway analysis of the differentially abundant proteins and metabolites for breast muscle

DA proteins and DA metabolites at stages ED 12 – ED 17, ED 17 – D 1 and D 1 – D 14 were subjected to integrative analysis within each breed (Additional file 2: Tables S2, and Additional file 3: Table S3). Results were focused on significant pathways related to at least two developmental stages.

**For the BJY chickens**, there were more than two DA proteins and metabolites participating in 14 significant pathways (Table 4). Six pathways were enriched during the entire interval (ED12-D14) examined in the current study, including FXR/LXR/RXR Activation, Actin Cytoskeleton Signaling, TCA Cycle II.

**Table 4:** The pathways and related differentially abundant proteins and metabolites for breast muscle in Beijing-You chickens^a^

PATHWAY	ED 12 - ED 17	ED 17 - D 1	D 1 - D 14
FXR/RXR Activation, LXR/RXR Activation	SERPINA1, COL3A1, RBP4, androsterone	APOB, C3, APOA1, VTN, GC	KNG1, C3, APOA1, TF, VTN, AHSG, GC, androsterone
Calcium Signaling	MYH2, TNNI2, TPM1, MYH7B, MYL1, CASQ2	MYH10, MYH2, MYH9, TNNC2, TNNT3, ATP2A2	MYH2, acetylcholine, TNNC1
Actin Cytoskeleton Signaling, Tight Junction Signaling	MYH2, MYL10, FLNA, ACTN2, MYH7B, MYL1	MYH10, MYH2, MYH9, ACTN2	KNG1, MYH2, acetylcholine, F2
Clathrin-mediated Endocytosis Signaling	HSPA8, SERPINA1, RBP4	SH3GL1, APOB, APOA1	APOA1, TF, F2
Unfolded protein response, Protein Ubiquitination Pathway	HSPA8, HSPA4, HSPA9, HSPA2, HSP90AA1, FKBP4	HSP90B1, HSPA5	P4HB, VCP
TCA Cycle II (Eukaryotic)	ACO2, succinic acid	SDHA, SUCLA2, L-malic acid, ACO2, IDH3A, MDH2, OGDH, succinic acid	L-malic acid, succinic acid
Protein Kinase A Signaling	GNB1, prostaglandin E2, MYH2, MYL10, TNNI2, FLNA, RACK1, MYL1	–	prostaglandin E2,MYH2, YWHAE, PDIA3, YWHAZ, PPP1R3A, PHKG1
Purine Nucleotides Degradation	inosine, hypoxanthine,	–	guanine, inosine, hypoxanthine, guanosine
Acute Phase Response Signaling	–	PLG, C3, APOA1	PLG, C3, APOA1, TF, AHSG, F2
Glycolysis I/ Gluconeogenesis I	–	PGK1, L-malic acid, MDH2	PGK1, GPI, L-malic acid, PGAM1, ENO2, GAPDH, BPGM, FBP2
PPARγ/RXR Activation	–	HSP90B1, ACADL, APOA1, PDIA3, GOT2	ACADL, APOA1, PDIA3
Superpathway of Methionine Degradation	–	BHMT, MAT1A, GOT1, GOT2	betaine, AHCY
Coagulation System	–	PLG, SERPINC1	KNG1, PLG, F2
Hypoxia Signaling in the Cardiovascular System	–	HSP90B1, LDHA	P4HB, LDHA

^a^-means no differentially abundant proteins and metabolites enriched

Different from the Cobbs, seven proteins related to Unfolded protein response and Protein Ubiquitination Pathway, including HSPA8, HSPA4, HSPA9, HSPA2, HSP90AA1, FKBP4, were enriched specifically in embryonic BJY and this pathway might play an important role from ED 12 until D 14. Six pathways were enriched during ED 17 to D 14, including Glycolysis I/Gluconeogenesis I and PPARγ/RXR Activation. It is worth noting that five proteins enriched in PPARγ/RXR Activation started to express differentially as early as ED 17, including ACADL, which might contribute to the fast accumulation of embryonic IMF. Pathways related to Purine metabolism were enriched as well.

**Fig. 6:**
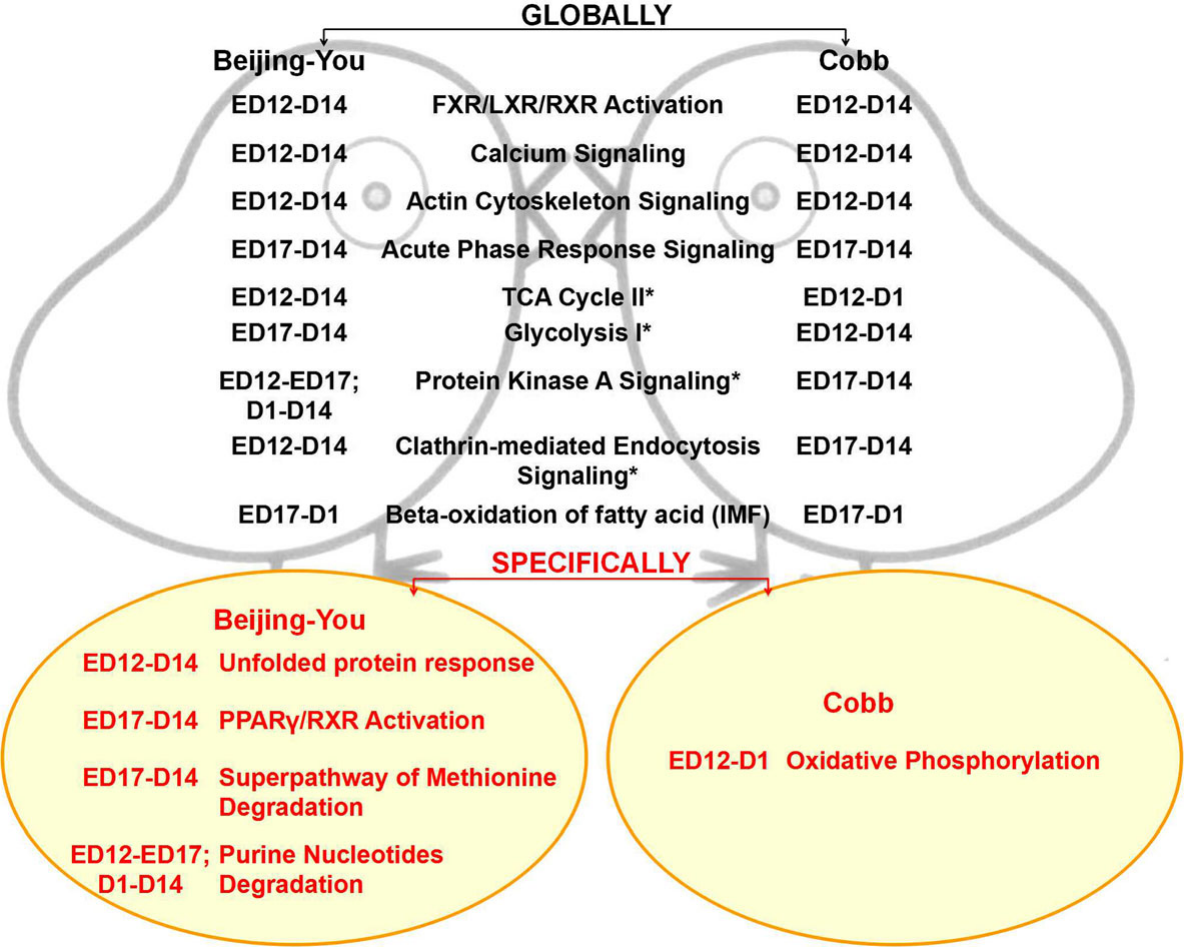
Global and breed-specific pathways for early muscle and IMF development in local BJY and commercial Cobb chickens. Stage information for each enriched pathway was given where ED 12 means embryonic day 12; ED 17 means embryonic day 17; D 1 is the hatching day and D 14 is the day 14 post-hatching; symbol * means the pathway was enriched at different stages in different breeds; key proteins and metabolites which mediated each pathway can be found in Tables 4 and 5; Beta-oxidation (mitochondria) of fatty acid pathway was enriched for IMF accumulation in both breeds

**For Cobb chickens,** there were two to eight DA proteins and metabolites participating in 10 significant pathways (Table 5). Four pathways were enriched throughout (ED 12 – D 14), including FXR/LXR/RXR Activation, Calcium Signaling and Glycolysis I/Gluconeogenesis I. Of special interest, as many as seven DA proteins and metabolites, viz. NDUFS1, ATP5B, UQCRC2, ACO2, VDAC1,VDAC2 and palmitic acid, were enriched in Oxidative Phosphorylation/Mitochondrial Dysfunction pathway during the embryonic stages studied; these might play roles in the rapid muscle growth (8.4 fold change from ED 12 – ED 17) and increase in fiber density from ED 17 – D 1. Global and breed-specific pathways for muscle development in local BJY and commercial Cobb chickens are indicated in Fig. 6.

**Table 5:** The pathways and key proteins and metabolites for breast muscle in Cobb chickens^a^

PATHWAY	ED 12 - ED 17	ED 17 – D 1	D 1 - D 14
FXR/RXR Activation, LXR/RXR Activation	APOB, SERPINA1, COL3A1, FGA	LYZ, APOA1, APOH, androsterone	ALB, TF, APOH, GC, androsterone
Calcium Signaling	MYH2, TNNI2, acetylcholine, TPM1, CASQ2, ACTA1	TNNI2, TNNC2, TNNC1, TPM1, ATP2A2, CASQ2	MYH2, acetylcholine, TNNC1
Actin Cytoskeleton Signaling	TLN2, MYH2, MYL10, acetylcholine, TLN1, ACTA1	FN1, FLNA, ACTN2	MYH2, acetylcholine, ARPC3, GSN
Glycolysis I/ Gluconeogenesis I	ENO1, MDH2	PGAM1, MDH1, MDH2	PGK1, GPI, PGAM1, GAPDH, FBP2
TCA Cycle II (Eukaryotic)	ACO2, DLD, FH, MDH2	ACO2, MDH1, FH, MDH2, OGDH	–
Oxidative Phosphorylation/ Mitochondrial Dysfunction	NDUFS1, ATP5B, UQCRC2, ACO2, VDAC1, palmitic acid, VDAC2	ATP5B, ACO2, NDUFB6, OGDH, palmitic acid, ATP5F1, VDAC2	
Hepatic Fibrosis / Hepatic Stellate Cell Activation	MYH2, COL6A1, COL6A2, COL20A1, COL3A1	–	MYH2, COL4A1, COL12A1
Acute Phase Response Signaling	–	FN1, APOA1, APOH	ALB, TF, APOH
Protein Kinase A Signaling	–	prostaglandin E2, TNNI2, FLNA, PDIA3, PYGB	prostaglandin E2, PHKB, MYH2, YWHAE, YWHAZ, PYGB
Clathrin-mediated Endocytosis Signaling	–	SH3GL1, LYZ, APOA1	ALB, TF, ARPC3

^a^-means no differentially abundant proteins and metabolites enriched

Although large differences exist between two breeds at different stages in terms of growth rate or developmental process, it supplied good chance to explore common signatures at molecule level for muscle development in chicken. The common proteins or metabolites involved in common pathways for both breeds were focused.

There were eight DA proteins and two DA metabolites that might be necessary for muscle development during the middle embryonic period (ED 12 - ED 17), including MYH2, TPM2 and inosine (Table 6). The 14 DA proteins and three DA metabolites for muscle during the late embryonic period (ED 17 – D 1), included ACADL, ATP5bB, and palmitic acid (Table 7); finally 13 DA proteins and five DA metabolites for muscle were identified during early post-hatch growth (D 1 – D 14), included GAPDH and acetylcholine (Table 8).

**Table 6:** Important proteins and metabolites playing roles in muscle development during the embryonic period (ED 12 - ED 17)^a^

ID	Protein/Compound Name	Gene Name	BJY Fold change	Cobb Fold change	Pathway
gi|45,383,668	Myosin-3	MYH2	11.52	8.47	Actin Cytoskeleton Signaling
gi|148,225,102	Myosin Regulatory light chain 2B	MYL10	0.33	0.21	Actin Cytoskeleton Signaling
gi|478,246,981	Serpin peptidase inhibitor, clade A, member 4 precursor	SERPINA1	0.33	0.50	FXR/RXR Activation
gi|206,597,436	Collagen alpha-1(III) chain precursor	COL3A1	7.05	28.30	FXR/RXR Activation
gi|45,383,738	Aconitate hydratase	ACO2	4.76	4.53	TCA cycle
gi|45,382,253	Troponin I, fast skeletal muscle	TNNI2	18.83	20.34	Calcium Signaling
gi|45,382,323	Tropomyosin alpha-1 chain	TPM1	15.07	99.08	Calcium Signaling
gi|46,093,996	Calsequestrin-2 precurso	CASQ2	9.42	6.96	Calcium Signaling
HMDB00195	inosine	–	18.00	49.86	Purine Nucleotides metabolism
HMDB00292	hypoxanthine	–	8.57	22.47	Purine Nucleotides metabolism

^a^- means no differentially abundant proteins and metabolites a a enriched

**Table 7:** Important proteins and metabolites playing roles in muscle development during the late embryonic period (ED 17 – D 1)

ID	Protein/Compound name	Gene name	BJY (FC)	Cobb (FC)	Pathway
gi|45,382,961	Apolipoprotein A-I preproprotein	APOA1	3.05	3.55	FXR/RXR Activation
gi|45,384,348	Aspartate Aminotransferase, cytoplasmic	GOT1	3.04	4.09	Amino acid metabolism
gi|45,383,117	Endophilin-A2	SH3GL1	0.39	0.39	Endocytosis Signaling
gi|448,261,627	Mitochondrial ATP Synthase Beta Subunit	ATP5B	2.96	2.51	Oxidative Phosphorylation
gi|363,743,079	ATP synthase subunit b, mitochondrial	ATP5F1	3.04	1.72	Oxidative Phosphorylation
gi|45,383,738	Aconitate hydratase	ACO2	3.00	3.13	Oxidative Phosphorylation
gi|45,383,562	Endoplasmin precursor	HSP90B1	0.39	0.43	PPARγ/RXR Activation
gi|57,529,797	Long-chain specific acyl-CoA dehydrogenase, mitochondrial	ACADL	2.15	2.30	PPARγ/RXR Activation
gi|45,383,890	Protein disulfide-isomerase A3 precursor	PDIA3	0.48	0.49	PPARγ/RXR Activation
gi|50,758,110	Malate dehydrogenase, mitochondrial	MDH2	4.21	1.59	TCA Cycle
gi|71,897,293	2-oxoglutarate dehydrogenase, mitochondrial	OGDH	3.12	2.72	TCA Cycle
gi|45,382,067	Troponin C, skeletal muscle	TNNC2	2.35	2.35	Calcium Signaling
gi|430,736,679	Endoplasmic reticulum calcium ATPase 2 isoform 1	ATP2A2	3.03	4.58	Calcium Signaling

**Table 8:** The differentially abundant proteins and metabolites playing roles in muscle development during early post-hatchling growth (D 1 – D 14)^a^

ID	Protein/Compound name	Gene	BJY (FC)	Cobb (FC)	Pathway
gi|45,384,486	Phosphoglycerate kinase	PGK1	3.29	14.51	Glycolysis I
gi|57,524,920	Glucose-6-phosphate isomerase	GPI	6.87	5.49	Glycolysis I
gi|45,382,061	Triosephosphate isomerase	TPI1	5.93	5.14	Glycolysis I
gi|71,895,985	Phosphoglycerate mutase 1	PGAM1	12.89	7.46	Glycolysis I
gi|46,048,961	Glyceraldehyde-3-phosphate dehydrogenase	GAPDH	1.76	1.93	Glycolysis I
gi|347,800,728	6-phosphofructokinase	PFKM	7.45	8.14	Glycolysis I
gi|50,762,391	Fructose-1,6-bis phosphatase isozyme 2	FBP2	17.10	11.04	Glycolysis I
gi|45,382,425	vitamin D-binding protein precursor	GC	0.18	0.19	FXR/RXR Activation
HMDB00031	androsterone	–	0.034	1.28E-07	FXR/RXR Activation
gi|45,384,092	Troponin C, slow skeletal and cardiac muscle	TNNC1	22.57	22.67	Calcium Signaling
HMDB00895	acetylcholine	–	39.12	24.42	Calcium Signaling
gi|55,741,616	14–3-3 protein epsilon	YWHAE	0.25	0.16	Protein Kinase A Signaling
gi|71,897,035	14–3-3 protein zeta	YWHAZ	0.21	0.20	Protein Kinase A Signaling
gi|513,217,491	Adenosylhomocysteinase	AHCY	0.18	0.16	Methionine Degradation
gi|45,385,813	Ovotransferrin precursor	TF	0.17	0.21	Acute Phase Response Signaling
HMDB00254	succinic acid	–	5.98	40.80	TCA Cycle II
HMDB00062	L-carnitine	–	7.16	19.03	L-carnitine Biosynthesis
HMDB00220	palmitic acid	–	1.68	2.28	Mitochondrial Dysfunction/Stearate Biosynthesis I

^a^- means no differentially abundant proteins and metabolites a enriched

### Identification and pathway analysis of the differentially abundant proteins related to IMF

The major period of accumulation of IMF was from ED 17 to D 1 and it was then depleted dramatically between D 1 and D 14 (Figs. 3, and 4). DA proteins and metabolites with exactly consistent changing pattern with the changes in IMF across these two stages were found and proposed as being potentially important molecules for IMF. Eight proteins were significantly altered in abundance between ED 17 and D 1 with reciprocal changes occurring from D 1 to D 14 in both breeds (Table 9). For example, abundant of several rate-limiting enzymes for mitochondrial beta-oxidation of fatty acid, ACADL, ACAD9, HADHA, HADHB, were increased before D1 and decreased from D1 to D14 (Fig. 6). Additionally, the changing pattern of the cholesterol transfer protein (APOA1), muscle related proteins (LAMA2 and TPM2) and calcium-binding protein (SRL) were consistent or opposite with those of IMF in both breeds.

**Table 9:** The differentially abundant proteins and metabolites playing roles in IMF

ID	Protein name	Gene name	BJY (FC)^a^	Cobb (FC)
gi|57,529,797	Long-chain specific acyl-CoA dehydrogenase	ACADL	2.15/0.35	2.29/0.35
gi|45,382,961	Apolipoprotein A-I preproprotein	APOA1	3.05/0.03	3.55/0.05
gi|45,384,238	Trifunctional enzyme subunit alpha	HADHA	2.20/0.32	2.17/0.21
gi|513,179,326	Trifunctional enzyme subunit beta	HADHB	3.28/0.29	1.80/0.35
gi|57,524,955	Acyl-CoA dehydrogenase family member 9, mitochondria	ACAD9	2.39/0.31	2.71/0.34
gi|45,384,504	Sarcalumenin precursor	SRL	2.33/0.36	2.17/0.21
gi|513,177,212	Laminin subunit alpha-2	LAMA2	2.03/0.45	2.45/0.29
gi|45,382,083	Tropomyosin beta chain	TPM2	2.35/0.25	2.56/0.34

^a^Fold change between ED 17 – D 1 and / that between D 1 – D 14 are shown

### Validation of DA proteins with western blotting

**Fig. 7:**
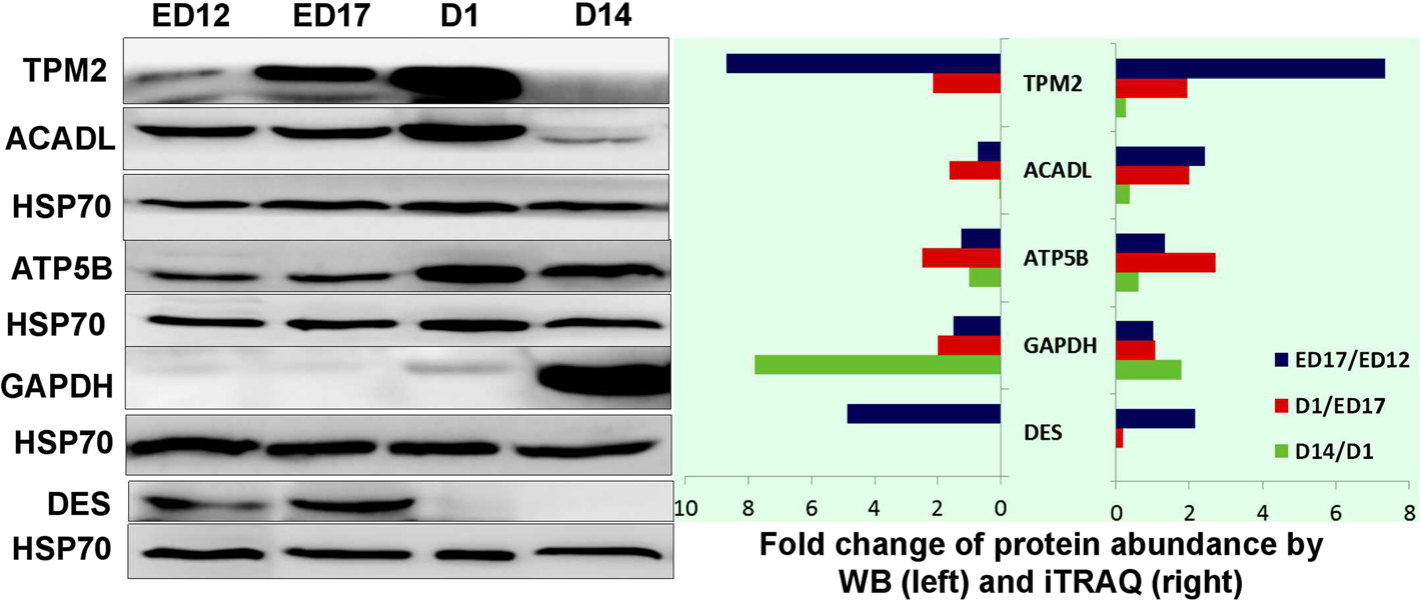
Comparison between levels of TPM2, ACADL, ATP5B, GAPDH, DES determined by Western blotting and iTRAQ in BJY. HSP70 was used as the reference protein

**Fig. 8:**
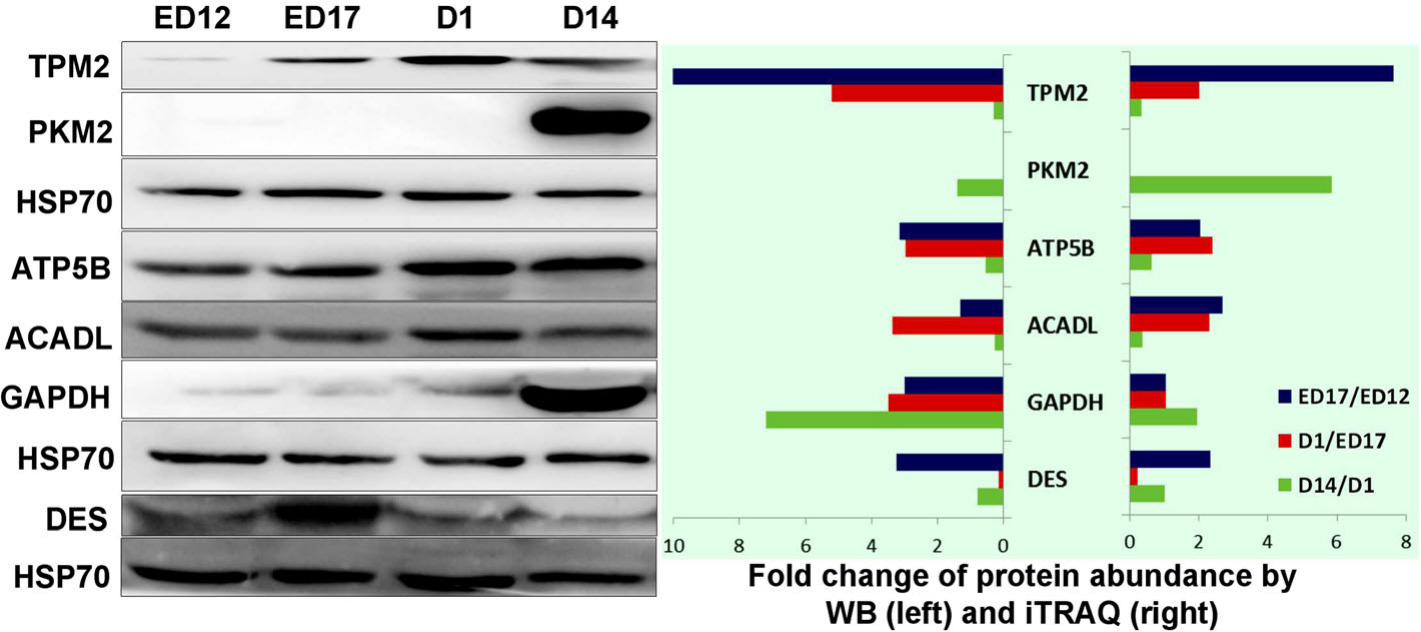
Comparison between levels of TPM2, PKM2, ATP5B, ACADL, GAPDH, DES determined by Western blotting and iTRAQ in Cobb. HSP70 was used as the reference protein

Western blotting of several proteins was used to verify the DA protein profiles obtained by the iTRAQ analyses. Selection was based on the biological functions and the protein profiles. Four proteins (PKM2, GAPDH, DES and ATP5B) were candidates for muscle development; two proteins (ACADL and TPM2) were related to IMF, and the rest were selected randomly. The results showed acceptable consistency between the quasi-quantitative Western blots and the fold-changes of DA proteins from the iTRAQ analysis (Figs. 7 and 8).

## Discussion

Chickens are among the most important food animals of worldwide economic significance and are widely used as a model species for the study of embryonic development. To our knowledge, no study has systematically examined the proteomes and metabolomes of embryonic skeletal muscle and IMF development.

In the current study, obvious phenotypic patterns exist both in slow-growing BJY and fast-growing Cobb chickens which are in line with the three critical stages in muscle development: ED 12 to ED 17 (embryonic rapid growth, hyperplasia), ED 17 to D 1 (hyperplasia and embryo preparing for hatch), D 1 to D 14 (shift from myoblast-mediated growth to satellite cell-mediated growth by hypertrophy) [5, 6, 14].

For muscle development, interest centered on the striking divergence of slow-growing and fast-growing birds and the underlying molecular basis for it. Breast muscle mass during middle-embryo and post-hatch stages was dramatically increased, which was in accordance with that observed in previous reports [15, 16]. In the BJY with no history of genetic selection for pectoral muscle size, the muscle weight increased 1.8 fold during the hatchling period accompanied by increasing fiber diameter and fiber cross-sectional area (could not be measured for ED 12 reliably). The fiber diameter and fiber cross-sectional area decreased from ED 17 – D 1 in Cobb. Correspondingly, the fiber density increased marginally from ED 17 – D 1 in Cobb but decreased significantly in BJY. That indicated the growth of the embryonic muscle weight in Cobb is mainly a result of stronger hyperplasia process instead of hypertrophy, which contribute to the post-hatchling excellent muscle mass achievements in fast-growing Cobb (35.9 fold change from D 1 to D 14).

For deposition of IMF in chicken embryo, this study is the first to show that the major time for accumulation of embryonic IMF was from ED 17 to D 1, the period when muscle growth was the least; the striking muscle growth occurred after D 1 (D 1 – D 14). These observed phenotypic patterns were supported by the proteomic and metabolomic profiles of breast muscle evident at ED 12, ED 17, D 1 and D 14 in the two breeds.

For proteomic profiling, mass spectrometry (MS)-based proteomics were applied by the 8-plex iTRAQ strategy, where each plex using two protein pools (*n* = 5 individuals). Pooling strategy with two biological replicates was applied, as suggested by Gan et al., (2007), which has the potential advantage of decreasing overall variability due to differences between individuals [17]. The correlation between DA fold changes for proteins in the two replicates were all high (R^2^ > 0.94, Additional file 4: Figures S1, and S2). The consistency between the quasi-quantitative Western blots and the changing pattern of DA proteins from the iTRAQ analysis were acceptable. As metabonomics reflects, in part, the downstream consequence of proteomics, it is also regarded as the complement of other “omics” for interpretation of functional genomics [18]. For metabolomic profiling in the current study, ten biological replicates for each age were from the same batch of chickens used for proteomic profiling. The advantage of combined proteomic and metabolomic profiling is that more profound results could be acquired than independent proteome analysis.

Through analysis of DA proteins and metabolites in the context of development, a remarkable finding is that, for embryonic Cobbs, many DA molecules (NDUFS1, ATP5B, UQCRC2, ACO2, VDAC1, VDAC2 and palmitic acid) were concentrated in Oxidative Phosphorylation/Mitochondrial Dysfunction pathway; those are vital for energy generation and metabolism (Tables 5, 7, 8) [19–22]. The network analyses (Additional file 4: Figure S3) were consistent with the energy requirement being greater for the faster muscle growth of the Cobbs. ATP5B is the catalytic subunit of the rate-limiting enzyme of oxidative phosphorylation and its abundance increased from ED 12 – ED 17 in Cobbs, as shown by Western blotting (Fig. 7).

Different from the Cobbs, in BJY, numerous proteins related to protein processing (HSPA8, HSPA4, HSPA9, HSP90AA1 and FKBP4) showed highest abundance at ED 12 and decreased thereafter (Additional file 4: Figure S4). As chaperones, heat shock proteins are known to aid in the folding of newly synthesized proteins and have roles in the disaggregation and degradation of persistently misfolded proteins [23]. The similar changing pattern of molecular abundance in Purine metabolism pathways (inosine and xanthine), indicated that genetic information processing might be more active in the embryonic development of slow-growing breeds. Findings from this study, especially on the metabolites in the faster growing Cobbs, might supply extra evidence for Oliveira’s hypothesis that high yield meat birds may direct more caloric resources towards muscle growth than towards fueling the hatching process [14].

Specific for BJY chickens, a series of DA proteins (ACADL, APOA1, HSP90B1, PDIA3 and GOT2) were enriched in PPAR signaling after ED 17 (Tables 4, 7, 8), among which the increase in ACADL, verified by Western blotting, changing in a manner that is consistent with those of IMF from ED 17 to D 14. Recent studies proved that PPARs functions as a key regulator for adipocyte development and IMF regulation [9, 24, 25]. Those proteins might contribute to the high capacity for accumulation of IMF in BJY chickens. The high abundances of ACADL at D 1 compared to post-hatchling days were consistent with previous reports [8, 26]. The most obvious fat accumulation at ED 17 – D 1 was also supported by the most significant IPA network constructed from DA proteins and metabolites between ED 17 and D 1, in which 29/34 proteins were detected and enriched (Additional file 4: Figure S5). It is worth noting the role of the methionine pathway in early muscle development. Key enzyme proteins for methionine degradation were decreased from ED17 to D 14, including MAT1A, BHMT or AHCY (Tables 4, 8). It is well established that MAT1A is the main gene responsible of S-adenosylmethionine (SAM) synthesis. Inhibition of AHCY can make S-adenosyl-homocysteine (SAH) accumulation, consequently suppressing the use of SAM for transmethylation reactions [27]. BHMT is a methyl transferase that can catalyze the transfer of the methyl group from betaine to homocysteine, which supplies the methyl group to methylate DNA [28]. A consistent finding in the present study was the decreased abundance of betaine from D 1 to D 14. Taken together, it indicated that the transmethylation reaction was attenuated from ED 17 to D 14 in BJY, which might contribute to the little overlap found in DA proteins between periods for pre- and post-hatching muscle growth (ED 12 - ED 17 vs D 1 - D 14). Considering that the abundance of AHCY and BHMT were also decreased in Cobb from D1 to D 14, further study is needed to clarify the epigenetic changes underlying altered protein abundance in early stages of chicken muscle and IMF development.

For the common features between the two breeds, several pathways, key proteins and metabolites (Tables 6, 7, 8) are known to be involved in muscle development. Some pathways, including FXR/RXR and LXR/RXR Activation, Calcium Signaling, Glycolysis I/Gluconeogenesis I, TCA Cycle II and Actin Cytoskeleton Signaling, were enriched in more than two stages. It was interesting that the abundance of androsterone, a weak androgen, decreased from D 1 to D 14 in both breeds. As an activator of the nuclear hormone receptor farnesoid X receptor (FXR) [29], the changing pattern is consistent with that of the FXR/RXR activation pathway, which was inactive from D 1 – D 14. Additionally, consistent with the increased protein abundance of TCA cycle enzymes, including ACO2 and/or MDH2, there were increases in L-malic acid (in BJY from ED 17 to D 14) and succinic acid (in both breeds from D 1 to D 14).

It is also worth noting the roles of the DA proteins enriched in Protein Kinase A Signaling in muscle development. Six to eight DA proteins and metabolites were enriched in that pathway in more than two stages in both breeds indicating the likelihood of their contributing to muscle glycogen metabolism. The breast muscle of the avian embryo is metabolically important mainly because of its large storage capacity for glycogen [6]; an important source of energy for skeletal muscle. The enriched proteins included YWHAE and YWHAZ, which belong to the 14–3-3 family of proteins, and were found to decrease from fast growing stages (D 1 - D 14) in both breeds. That is perhaps consistent with the report that YWHAE inhibited hypertrophy of cardiomyocytes [30]. Additional study of the function and mechanism of action of 14–3-3 proteins in development of skeletal muscle is needed. Acute Phase Response Signaling was also enriched in both breeds from ED 17 to D 14. The abundance of most of the enriched proteins, including APOA1 and APOH in Cobbs and PLG, C3 and APOA1 in BJY showed reciprocal changes between ED 17 – D 1 and D 1 – D 14 similar to that of IMF content. The function of complement C3 precursor has been reported to relate to insulin secretion and glucose homeostasis [31], but its possible relationship with IMF has not been described.

As for IMF accumulation, according to the findings in cattle, the level of IMF at the start of the growth period is likely a key determinant of the final level of IMF after finishing [32]. In chickens, the early period of change in IMF was observed between ED 17 and D1, when changes in muscle growth were least obvious. This suggested that myocytes and adipocytes influencing each other during development. The accretion rate of IMF depends on the number of adipocytes and their net accumulation of stored lipid. Aspects of muscle growth and its metabolic activity are also important. For example, animals having a high muscularity with a high glycolytic activity display a reduced development of IMF [2]. In the current study, several proteins altered in abundance between ED 17 and D 1 and reciprocal changes were demonstrated from D 1 to D 14 and this changing pattern was exactly consistent with the changes in IMF. Of special interest, four were rate-limiting enzymes for mitochondrial beta-oxidation of fatty acid (ACADL, ACAD9, HADHA, HADHB), which highlight the important role of this pathway playing in IMF accumulation at early stage. The ACADL catalyze the initial step of mitochondrial beta-oxidation of straight-chain fatty acid and its changes from iTRAQ analyses were verified by Western blotting. The results were consistent with previous report that lipid oxidation was very active from mid incubation until 2 or 3 days before internal pipping because adequate oxygen and yolk fatty acids could be supplied. Additionally, the abundant of apolipoprotein A1 was increased from D12 to D1, which might contribute to lipoproteins taking up cholesterol from the yolk sac membrane [14]. Consistent with Cui’s report [9], genes and pathways related to muscle development and ECM were also involved in IMF deposition, including the muscle related protein LAMA2 [33], an extracellular protein TPM2 [34] and the calcium-binding protein SRL [35]. The changes in TPM2 from iTRAQ analyses were verified by Western blotting. As it is difficult to quantify IMF content during embryonic development in chickens, it emphasizes the utility of suitable bio-markers.

## Conclusion

In summary, this study found that ED 17 - D 1 was the earliest period for IMF accumulation. Through integrated analysis of the protein and metabolite profiles of breast muscle, pathways related to protein processing and PPAR signaling were found to be enriched to support high capacity of embryonic IMF accumulation in Beijing-You. Pathways related to ATP production were enriched to support the fast muscle growth in Cobb. Mitochondrial beta-oxidation of fatty acid is identified as the key pathway related to chicken IMF deposition at early stages.

## Methods

### Animals

The Beijing-You (BJY) and Cobb-Vantress (Cobb) eggs were obtained from the experimental farm of the IAS (CAAS, Beijing, China) and the Anhui five-star cultivation group CO. LTD (Ningguo, China). All eggs were incubated with the normal procedure and chicks were reared in caging under continuous lighting using standard conditions of temperature, humidity and ventilation at the farm of the IAS, CAAS. Chickens used for sample collection at D 1 were not fed. The same diet was fed to all chickens and was formulated to be intermediate between recommendations for the two breeds [36, 37]. The starter ration (D 1 to D 21) provided 20% crude protein and 2.87 MCal/kg energy. Feed and water were provided ad libitum*.*

### Tissue sampling

Breast muscles were collected from embryos at ED 12 and ED 17 or chicks of similar weights at D 1 and D 14 within each breed. Chicks were electrically stunned and killed by exsanguination. Because of the limited amount of sample from each bird, especially during the embryonic stages, 10 embryos or birds with similar egg/body weight were used for protein analysis, an additional 10 for histology and 10 for metabolomic analysis. The embryos, bodies and breast muscles on both sides were weighed. Sub-samples were immediately fixed in 4% paraformaldehyde and held at room temperature and additional samples were snap frozen in liquid nitrogen and held at −80 °C. The latter samples were used for the iTRAQ, UHPLC–MS, Western blotting and oil red O staining.

### Histology

Two or three serial cross-sections of five randomly selected chickens per age in the two breeds were used to evaluate the area, diameter and density of muscle fibers (stained with hematoxylin and eosin) and oil red O staining. Fixed tissues were dehydrated through an ascending ethanol series, embedded in paraffin, and sectioned (3–5 μm). After dewaxing in xylene and rehydration using a descending alcohol gradient, mounted muscle sections were stained with hematoxylin and eosin (H&E). Oil red O staining was as follows: frozen sections (4–8 μm) were air dried for 15–20 min then immersed in 100% isopropanol for 5 min, 0.5% working solution of oil red O for 7–8 min then 85% isopropanol for 3 min. Thereafter, sections were washed with three exchanges of deionized water and counterstained with hematoxylin for 1–1.5 min to visualize nuclei. Sections were rinsed with running tap water for 10 min and covered with a coverslip using 10% glycerol in PBS. Images were captured and processed with Image-Pro Plus 6.0 software.

### Protein extraction and quantitation

The muscle samples obtained at four ages from the two breeds were ground to powder in liquid nitrogen and dissolved in lysis buffer (9 M urea, 4% CHAPS, 1% DTT, 1% IPG buffer) at 30 °C for 1 h. The supernatants, obtained by centrifugation at 15,000 *g* for 15 min at room temperature, were re-centrifuged. Protein concentrations of the supernatants were then determined by the Bradford method. For each age and breed, two pools were constructed using equal amounts of protein from five of the 10 sampled individuals. Each pool was then diluted to the same concentration with TBS for iTRAQ labeling and then stored at −80 °C until analysis.

### Protein digestion and iTRAQ labeling

The two protein pools, representing biological replicates at each stage and breed, were used to determine the protein profiles. After precipitation with acetone, protein pellets were dissolved in the buffer from the iTRAQ kit (Sigma, St Louis, MO). Each sample was then reduced, alkylated, digested with trypsin, and labeled with the iTRAQ reagents as follows: each protein pellet for eight samples was reduced with 4 μL reducing reagent, incubated at 60 °C for 1 h, alkylated with 2 μL cysteine blocking reagent, and incubated at room temperature for 10 min. Trypsin digestion used 2.5 μg sequencing-grade trypsin (Promega, Madison, WI) and incubating the samples at 37 °C for 12 h. The peptides were precipitated and collected by centrifugation at 15,000 *g* for 20 min, then dissolved in 50 μL dissolution buffer and re-centrifuged.

The labeling was 2-plex for the two pools at each age and breed. For BJY, reporter tags 117/118, 115/116, 113/114, 119/121 were used for samples from ED 12, ED 17, D 1 and D 14, respectively. For the Cobbs, iTRAQ labeling used 115/116, 113/114, 119/121, 117/118 for samples from ED 12, ED 17, D 1 and D 14, respectively. The labeled peptide samples were pooled within each breed (8-plex, 100 g total peptide) and fractionated by strong cationic exchange (SCX) chromatography.

### 2D–LC-MSMS analysis

Chromatographic separation of the pooled samples was performed on an Agilent 1200 HPLC system (Agilent Technologies Inc., Santa Clara, CA). Labeled peptides were fractionated by strong cation exchange liquid chromatograph (SCX) using a 2.0 × 150 mm, 5 μm, 300 Å column (Michrom, Auburn, CA). The samples were dissolved in 100 μL buffer A (10 mM ammonium formate (pH = 2.8) with 20% ACN). Separation was performed at 0.3 ml/min using a nonlinear binary gradient from buffer A to 50–100% buffer B (500 mM ammonium formate (pH = 2.8) with 20% ACN) over 40 min, and 30 to 100% buffer B for 5 min. A total of 12 fractions were collected. The first fraction was collected from 0 to 5 min, and then 4 min fractions were collected from 6 to 44 min, and a final fraction from 45 to 50 min. Each fraction was vacuum freeze-dried for LC-MSMS analysis.

### RPLC-MSMS analysis

Fractions were dissolved in Nano-RPLC Buffer A (0.1% formic acid, 2% ACN). The online Nano-RPLC was employed on the Eksigent nanoLC-Ultra™ 2D System (SCIEX, Framingham, MA). The samples were loaded on a C18 nanoLC trap column (100 μm × 3 cm, C18, 3 μm, 150 Å) and washed with Nano-RPLC Buffer A (0.1% formic acid, 2% ACN) at 2 μL/min for 10 mins. Elution used a gradient of 5% to 35% Nano-RPLC buffer B (0.1% formic acid, 98% ACN) over 70 min using an analytical ChromXP C18 column (75 μm × 15 cm, C18, 3 μm 120 Å) with spray tip. Data was acquired by the Triple TOF 5600 System (SCIEX) fitted with a Nanospray III source (SCIEX) and a pulled quartz tip as the emitter (New Objective, Woburn, MA). The ion spray voltage of the mass spectrometer was set to 2.5 kV, the curtain and nebulizer gases were set to 30 psi and 5 psi, respectively, and the heated capillary temperature was set to 150 °C. Survey scans were acquired in 250 ms using the information dependent acquisition. Up to 35 product ion scans were collected if they exceeded 150 counts per second (counts/s) with a 2^+^ − 5^+^ charge-state. The total cycle time was fixed at 2.5 s. For collision-induced dissociation, a rolling collision energy setting was used to all precursor ions. A dynamic exclusion time was set for 1/2 of peak width (18 s). The precursor was then refreshed off the exclusion list.

### Differential abundance (DA) protein identification and quantification

The raw peptide, protein identification and quantification were performed using Protein Pilot v4.0 (SCIEX) against the chicken database with the Paragon algorithm [38]. Search parameters were set as follows: instrument was TripleTOF 5600, iTRAQ 8-plex quantification, trypsin as enzyme, fixed modification of cysteine by iodoacetamide, biological modification as the ID focus, thorough identification search. To reduce false positive identification, false discovery rate (FDR) less than 1% was required for all reported proteins by PSPEP (Proteomics System Performance Evaluation Pipeline Software, integrated into the Protein Pilot software). Proteins with > |1.5| -fold differences between coupled samples and *P*-value of less than 0.05 were determined as having differential abundance.

### Metabolomic sample preparation and quantitation

Breast muscle samples from 10 birds in BJY and Cobb at four different ages were prepared by powdering the tissue in liquid nitrogen. Sample (0.1 g) was mixed with 500 μL of 50% aqueous acetonitrile (Merck, Darmstadt, Germany) and centrifugation at 12,000 *g* and 4 °C for 15 min to eliminate the proteins. Supernatant (200 μL) was removed and diluted with 100 L acetonitrile (Merck, Darmstadt, Germany), then vortexed for 5 min. After centrifuging at 12,000 *g* and 4 °C for 10 min, the clear supernatant was transferred to the sampling vial and an aliquot of 80 μL was injected for UHPLC–MS analysis.

### UHPLC-MS analysis

UHPLC-MS analysis was performed on a 1290 Infinity LC system equipped with 6520 Accurate-Mass Quadrupole Time-of-Flight (Q-TOF) mass spectrometer (Agilent). Chromatographic separations were performed at 40 °C on a Waters ACQUITY UPLC HSS T3 column (2.1 mm × 100 mm, 1.8 μm, Milford, MA).The mobile phase consisted of 0.1% formic acid - water (A) and 0.1% formic acid - ACN (B). The optimized elution started from 5% buffer B at 0–2 min and increased to 95% buffer B between 2 and 17 min, then held at 95% buffer B for 2 min, followed by re-equilibrating for 6 min. The flow rate was 0.4 mL/min and the injection volume was 4 μL. The autosampler was maintained at 4 °C.

#### LC/MS analysis

An electrospray ionization source (ESI) was applied in positive and negative mode. The optimized parameters were set up as follows: capillary voltage, 4 kV for positive mode and 3.5 kV for negative mode; nebulizer pressure, 45 psig (310.3 kPa); drying gas flow, 11 L/min; gas temperature, 350 °C; fragmentor voltage, 120 V; skimmer voltage, 60 V. Data were acquired in centroid mode from 50 to 1100 m/z.

#### DA metabolite identification

Each sample was represented by a total ion current chromatogram. Agilent MassHunter Qualitative software was applied to convert the UHPLC–MS raw data to common data format files. The program XCMS (http://enigma.lbl.gov/xcms-online/) was used for peak detection, RT alignment and peak integration to generate a visual data matrix. The data of each sample were loaded to SIMCA-P software (MKS Umetrics, Umea, Sweden) for partial least squares-discriminate analysis. Statistically significant differences between mean values of two groups were tested by Student T-test in SPSS 19.0. The differences were considered significant when VIP (Variable Importance in the Projection) value >1 and *P* < 0.05.

### Integrated analysis of DA proteins and metabolites with IPA analysis

Ingenuity Pathway Analysis (IPA; Ingenuity Systems, Redwood City, CA; https://www.qiagenbioinformatics.com/products/ingenuity-pathwayanalysis/) was performed to identify the molecular pathways and network based on DA proteins and metabolites. Metabolite names were converted to the Human Metabolome Database (HMDB) ID with MetaboAnalyst 3.0 (http://www.metaboanalyst.ca/). The list of protein IDs and HMDB IDs was imported into the online software package IPA to determine their canonical pathways and molecular networks. The IPA content version is 27,821,452 and Release Date at 2016–06-14. Analyses were performed with thresholds of *P* < 0.05; both direct and indirect relationships were considered.

### Verification of protein abundance by western blotting

Sliced muscle tissue of each of two pools of five embryos or chicks at each stage was lysed on ice in RIPA lysis buffer with 1 mM PMSF (phenylmethanesulfonyl fluoride, Beyotime, Shanghai, China) and quantified by the Pierce BCA Protein Assay Kit (Thermo Fisher Scientific, Shanghai, China). Samples containing 32 g of total protein were separated by electrophoresis on 12% (*w*/*v*) SDS-PAGE and transferred to a PVDF membrane (Millipore, Darmstadt, Germany) for 1.5 h at 200 mA. After blocking for 1 h with 5% (w/v) nonfat dry milk in TBST (0.01% (*v*/v) Tween 20 in Tris-buffered saline), the membrane was probed with the primary antibodies, indicated below, diluted in blocking buffer, overnight at 4 °C. After washing with TBST and TBS, the membrane was incubated with HRP-conjugated goat anti-rabbit IgG, (CWBIO, Beijing, China), diluted in blocking buffer at room temperature for 1 h. After thorough washing, immunoreactive proteins were visualized by chemiluminescence with images being captured with an ImageQuant LAS 4000 mini machine (GE, Fairfield, CT), and the signal density data were analyzed with Image-Pro Plus 6.0 software. The primary antibodies, all rabbit polyclonals, were raised against: Long-chain specific acyl-CoA dehydrogenase (ACADL, 1:1000, Sigma-Aldrich), ATP synthase subunit beta (ATP5B, 1:3000, Agrisera, Vännäs, Sweden), alpha 2 Macroglobulin (A2M,1:300, Bioss, Beijing, China), Tropomyosin beta chain (TPM2, 1:400, LifeSpan BioSciences, Seattle, WA), Desmin (DES, 1:300, Abcam, Cambridge, MA), Glyceraldehyde-3-phosphate dehydrogenase (GAPDH, 1:10,000, Abcam), Heat shock protein 70 (Hsp70, 1:2000, Abcam), Pyruvate kinase (PKM2, 1:1000, Thermo Fisher Scientific, Shanghai, China).

## Additional files

Additional file 1: Table S1. Results of protein quantification by iTRAQ (XLSX 1692 kb)

Additional file 2: Table S2. Differential abundance proteins between the sequential sampling stages (XLSX 99 kb)

Additional file 3: Table S3. Differential abundance metabolites between the sequential sampling stages (XLSX 135 kb)

Additional file 4: Figure S1. Correlation of iTRAQ Log2 Ratio of differential abundant proteins between two biological replicates in BJY. **Figure S2.** Correlation of iTRAQ Log2 Ratio of differential abundant proteins between two biological replicates in Cobb. **Figure S3.** Top 1 significant network enriched by DA proteins and metabolites (from ED 12 – ED 17) related to Skeletal and Muscular Development in Cobb chickens. **Figure S4.** Top significant network enriched by DA proteins and metabolites (from ED 12 – ED 17) related to Protein Folding and Cell Morphology in BJY chickens. **Figure S5.** Top 1 Significant network (Top 1) enriched by DA proteins and metabolites (from ED 17 – D 1) related to Lipid Metabolism in BJY chickens (DOCX 1823 kb)

## Abbreviations

ACAD9: Acyl-CoA dehydrogenase family member 9, mitochondria
ACADL: Long-chain specific acyl-CoA dehydrogenase, mitochondrial
ACO2: Aconitate hydratase
AHCY: Adenosylhomocysteinase
APOA1: Apolipoprotein A-I preproprotein
APOH: Apolipoprotein H
ATP5B: Mitochondrial ATP Synthase Beta Subunit
BHMT: Betaine--Homocysteine S-Methyltransferase
BJY: Beijing-you chicken
C3: Complement C3
CAN: Acetonitrile
DA: Differential abundance
DES: Desmin
ED: Embryonic day
ESI: Electrospray ionization source
FDR: False discovery rate
FKBP4: FK506 Binding Protein 4
FXR: Farnesoid X receptor
GAPDH: Glyceraldehyde-3-phosphate dehydrogenase
GOT2: Glutamic-Oxaloacetic Transaminase 2
H&E: Hematoxylin and eosin
HADHA: Trifunctional enzyme subunit alpha
HADHB: Trifunctional enzyme subunit beta
HMDB: Human Metabolome Database
HSP90AA1: Heat Shock Protein 90 Alpha Family Class A Member 1
HSP90B1: Endoplasmin precursor
HSPA2: Heat Shock Protein Family A (Hsp70) Member 2
HSPA4: Heat Shock Protein Family A (Hsp70) Member 4
HSPA8: Heat Shock Protein Family A (Hsp70) Member 8
HSPA9: Heat Shock Protein Family A (Hsp70) Member 9
IMF: Intramuscular fat
IPA: Ingenuity Pathway Analysis
iTRAQ: Isobaric tags for relative and absolute quantitation
LAMA2: Laminin subunit alpha-2
LC-MS: Liquid chromatography/mass spectrometry
LXR: liver X receptor
MAT1A: Methionine Adenosyltransferase 1A
MDH2: Malate dehydrogenase, mitochondrial
MYH2: Myosin-3
Nano-RPLC: Nano-reversed-phase liquid chromatography
NDUFS1: NADH:Ubiquinone Oxidoreductase Core Subunit S1
PDIA3: Protein Disulfide Isomerase Family A Member 3
PKM2: Pyruvate Kinase, Muscle
PLG: Plasminogen
PPARγ: Peroxisome Proliferator Activated Receptor Gamma
PSPEP: Proteomics System Performance Evaluation Pipeline Software, integrated into the Protein Pilot software
RXR: Retinoid X receptor
SAH: S-adenosyl-homocysteine
SAM: S-adenosylmethionine
SCX: Strong cationic exchange
SRL: Sarcalumenin precursor
TPM2: Tropomyosin beta chain
UHPLC–MS: Ultra high performance liquid chromatography–tandem mass spectrometry
UQCRC2: Ubiquinol-Cytochrome C Reductase Core Protein II
VDAC1: Voltage Dependent Anion Channel 1
VDAC2: Voltage Dependent Anion Channel 2
VIP: Variable Importance in the Projection
YWHAE: 14–3-3 protein epsilon
YWHAZ: 14–3-3 protein zeta

## References

[1] Fernandez X, Monin G, Talmant A (1999). Influence of intramuscular fat content on the quality of pig meat. I. Composition of the lipid fraction and sensory characteristics of. Meat Sci.

[2] Hocquette JF, Gondret F, Baéza E (2010). Intramuscular fat content in meat-producing animals: development, genetic and nutritional control, and identification of putative markers. Animal.

[3] Devol DL, Mckeith FK, Bechtel PJ (1988). Variation in composition and palatability traits and relationships between muscle characteristics and palatability in a random sample of pork carcasses. J Anim Sci.

[4] Smith JH (1963). Relation of body size to muscle cell size and number in the chicken. Poult Sci.

[5] Sporer K R, Tempelman R J, Ernst CW , et al. Transcriptional profiling identifies differentially expressed genes in developing turkey skeletal muscle BMC Genomics, 2011, 12(1):143. 10.1186/1471-2164-12-143 PMC3060885 21385442

[6] Velleman SG (2007). Muscle development in the embryo and hatchling. Poult Sci.

[7] Chartrin P, Bernadet MD, Guy G (2007). Do age and feeding levels have comparable effects on fat deposition in breast muscle of mule ducks?. Animal.

[8] Liu J, Fu R, Liu R (2016). Protein profiles for muscle development and intramuscular fat accumulation at different post-hatching ages in chickens. PLoS One.

[9] Cui HX, Liu RR, Zhao GP (2012). Identification of differentially expressed genes and pathways for intramuscular fat deposition in pectoralis major tissues of fast-and slow-growing chickens. BMC Genomics.

[10] Ouyang H, Wang Z, Chen X, et al. Proteomic analysis of chicken skeletal muscle during embryonic development. Front Physiol. 2017;8(281) 10.3389/fphys.2017.00281 PMC5420592 28533755

[11] Zheng P (1988). Breeds of domesticated animal and poultry in China.

[12] China National Commission of Animal Genetic Resources (2010). Animal genetic resources in China- poultry.

[13] Liu R, Sun Y, Zhao G (2012). Genome-wide association study identifies loci and candidate genes for body composition and meat quality traits in Beijing-you chickens. PLoS One.

[14] Oliveira JED, Uni Z, Ferket PR (2008). Important metabolic pathways in poultry embryos prior to hatch. Worlds Poult. Sci. J..

[15] Zhu C, Gi G, Tao Z (2014). Development of skeletal muscle and expression of myogenic regulatory factors during embryonic development in Jinding ducks (Anas Platyrhynchos Domestica). Poult Sci.

[16] Guernec A, Berri C, Chevalier B (2003). Muscle development, insulin-like growth factor-I and myostatin mRNA levels in chickens selected for increased breast muscle yield. Growth Hormon IGF Res.

[17] Gan CS, Chong PK, Pham TK (2007). Technical, experimental, and biological variations in isobaric tags for relative and absolute quantitation (iTRAQ). J Proteome Res.

[18] Raamsdonk LM, Teusink B, Broadhurst D (2001). A functional genomics strategy that uses metabolome data to reveal the phenotype of silent mutations. Nat Biotechnol.

[19] Bénit P, Chretien D, Kadhom N (2001). Large-scale deletion and point muta-tions of the nuclear NDUFV1 and NDUFS1 genes in mitochondrial complex I defi-ciency. Am J hum genet. Am J Hum Genet.

[20] Herrmann AG, Deighton RF, Bihan TL (2013). Adaptive changes in the neuronal proteome: mitochondrial energy production, endoplasmic reticulum stress, and ribosomal dysfunction in the cellular response to metabolic stress. J Cereb Blood Flow Metab.

[21] Tang M, Liu BJ, Wang SQ (2014). The role of mitochondrial aconitate (ACO2) in human sperm motility. Syst Biol Reprod Med.

[22] Anflous K, Armstrong DD, Craigen WJ (2001). Altered mitochondrial sensitivity for ADP and maintenance of creatine-stimulated respiration in oxidative striated muscles from VDAC1-deficient mice. J Biol Chem.

[23] Hipp MS, Park SH, Hartl FU (2014). Proteostasis impairment in protein-misfolding and-aggregation diseases. Trends Cell Biol.

[24] Cipolletta D, Feuerer M, Li A (2012). PPAR-[ggr] is a major driver of the accumulation and phenotype of adipose tissue Treg cells. Nature.

[25] Lee HJ, Mi J, Kim H (2013). Comparative Transcriptome analysis of adipose tissues reveals that ECM-receptor interaction is involved in the depot-specific Adipogenesis in cattle. PLoS One.

[26] Doherty MK, Mclean L, Hayter JR (2004). The proteome of chicken skeletal muscle: changes in soluble protein expression during growth in a layer strain. Proteomics.

[27] Lee Y, Jeong LS, Choi S (2011). Link between allosteric signal transduction and functional dynamics in a multisubunit enzyme: S-adenosylhomocysteine hydrolase. J Am Chem Soc.

[28] Mato JM, Martínezchantar ML, Lu SC (2008). Methionine metabolism and liver disease. Annu Rev Nutr.

[29] Wang S, Lai K, Moy FJ (2006). The nuclear hormone receptor farnesoid X receptor (FXR) is activated by androsterone. Endocrinology.

[30] Liao W, Wang S, Han C (2005). 14-3-3 proteins regulate glycogen synthase 3beta phosphorylation and inhibit cardiomyocyte hypertrophy. FEBS J.

[31] Fiorentino TV, Hribal ML, Andreozzi F (2015). Plasma complement C3 levels are associated with insulin secretion independently of adiposity measures in non-diabetic individuals. Nutr. Metab. Cardiovasc. Dis..

[32] Pethick DW, Harper GS, Oddy VH (2004). Growth, development and nutritional manipulation of marbling in cattle: a review. Aust J Exp Agric.

[33] Colognato H, Yurchenco PD (1999). The laminin alpha2 expressed by dystrophic dy(2J) mice is defective in its ability to form polymers. Curr Biol.

[34] Marttila M, Lemola E, Wallefeld W (2012). Abnormal actin binding of aberrant beta-tropomyosins is a molecular cause of muscle weakness in TPM2-related nemaline and cap myopathy. Biochem J.

[35] Leberer E, Timms BG, Campbell KP (1990). Purification, calcium binding properties, and ultrastructural localization of the 53,000- and 160,000 (sarcalumenin)-dalton glycoproteins of the sarcoplasmic reticulum. J Biol Chem.

[36] NRC (National Research Council) (1994). Nutrient requirements of poultry.

[37] NY/T 33-2004 (Agriculture Standard of The People’s Republic of China) (2004). Feeding standard of chicken.

[38] Shilov IV, Seymour SL, Patel AA (2007). The paragon algorithm, a next generation search engine that uses sequence temperature values and feature probabilities to identify peptides from tandem mass spectra. Mol Cell Proteomics.

