# Supplementary material for: Uncovering the embryonic development-related proteome and metabolome signatures in breast muscle and intramuscular fat of fast-and slow-growing chickens
Source: BMC Genomics. 2017 Oct 23;18:816. doi: 10.1186/s12864-017-4150-3 (PMC5653991; doi:10.1186/s12864-017-4150-3)
Supplement: Supplementary file 4 — Correlation of iTRAQ Log2 Ratio of differential abundant proteins between two biological replicates in BJY. Figure S2. Correlation of iTRAQ Log2 Ratio of differential abundant proteins between two biological replicates in Cobb. Figure S3. Top 1 significant network enriched by DA proteins and metabolites (from ED 12 – ED 17) related to Skeletal and Muscular Development in Cobb chickens. Figure S4. Top significant network enriched by DA proteins and metabolites (from ED 12 – ED 17) related to Protein Folding and Cell Morphology in BJY chickens. Figure S5. Top 1 Significant network (Top 1) enriched by DA proteins and metabolites (from ED 17 – D 1) related to Lipid Metabolism in BJY chickens (DOCX 1823 kb) [file 12864_2017_4150_MOESM4_ESM.docx]

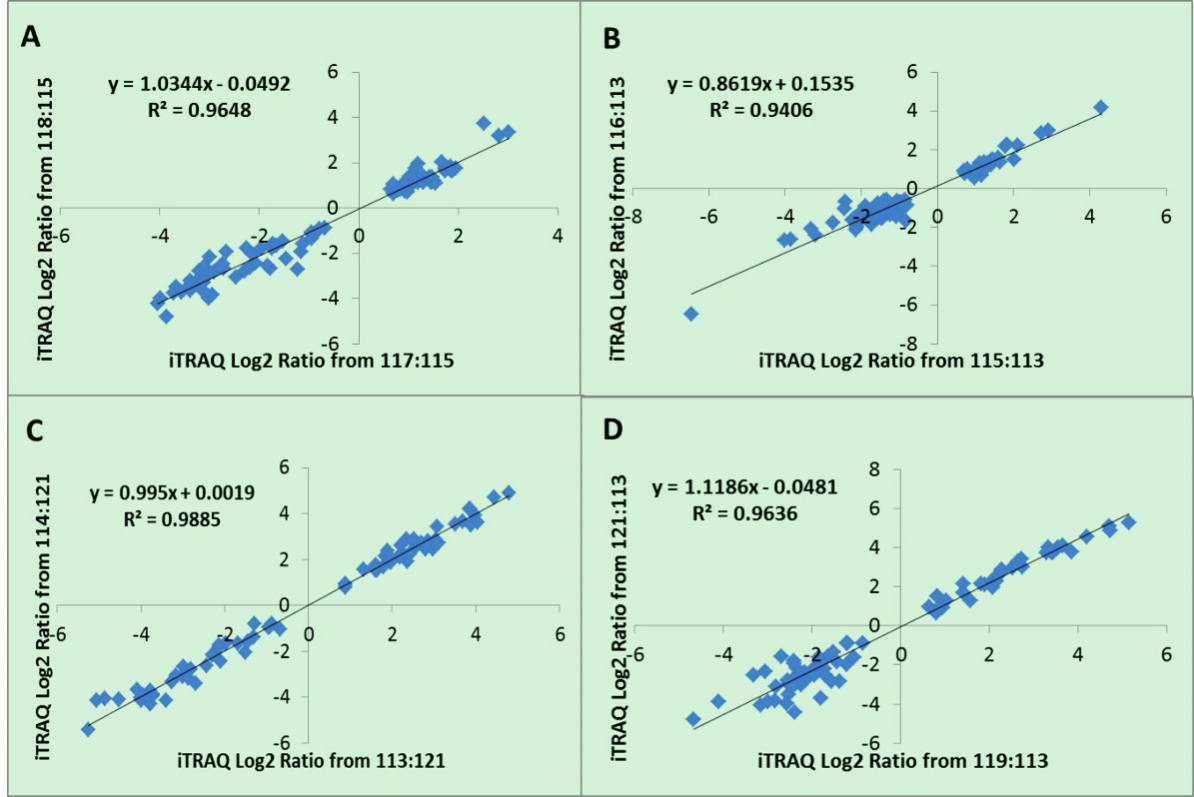


**Figure S1. Correlation of iTRAQ Log2 Ratio of differential abundant proteins between two biological replicates in BJY**: 117 and 118 for ED 12 (A, n = 92); 115 and 116 for ED 17 (B, n = 99); 113 and 114 for D 1 (C, n = 77); 119 and 121 for D 14 (D, n = 77).


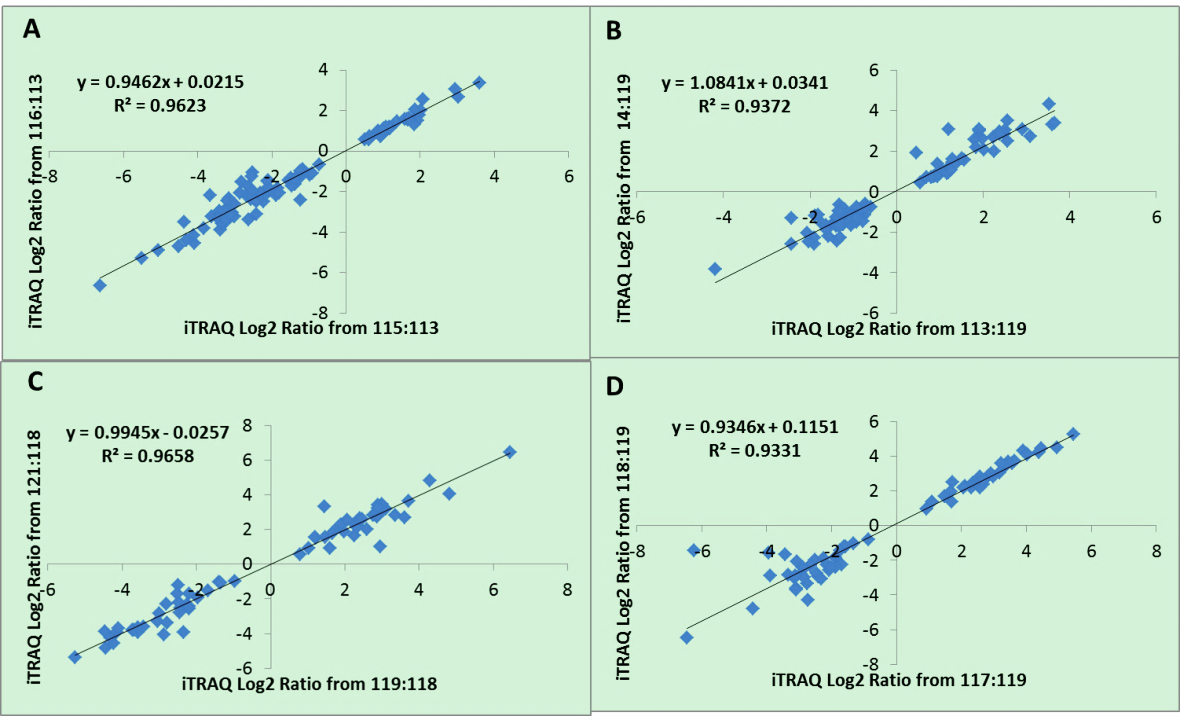


**Figure S2. Correlation of iTRAQ Log2 Ratio of differential abundant proteins between two biological replicates in Cobb**: 115 and 116 for ED 12 (A, n = 95); 113 and 114 for ED 17 (B, n = 92); 119 and 121 for D 1 (C, n = 68); 117 and 118 for D 14 (D, n = 68).


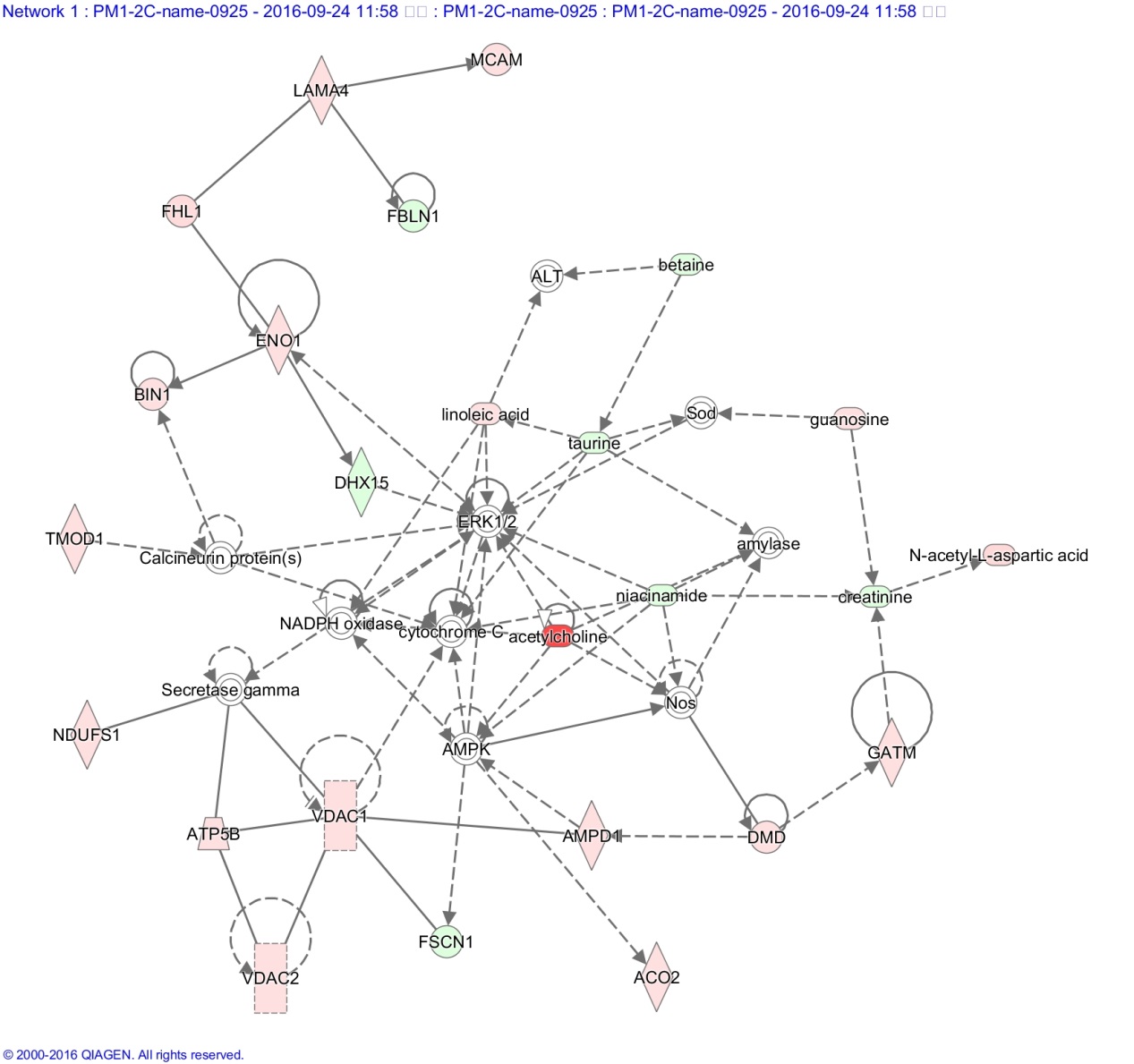


**Figure S3. Top 1 significant network enriched by DA proteins and metabolites (from ED 12 – ED 17) related to Skeletal and Muscular Development in Cobb chickens.** Red and green symbols show the up-regulated and down-regulated molecules found in the current study while white symbols indicate genes that are functionally associated.


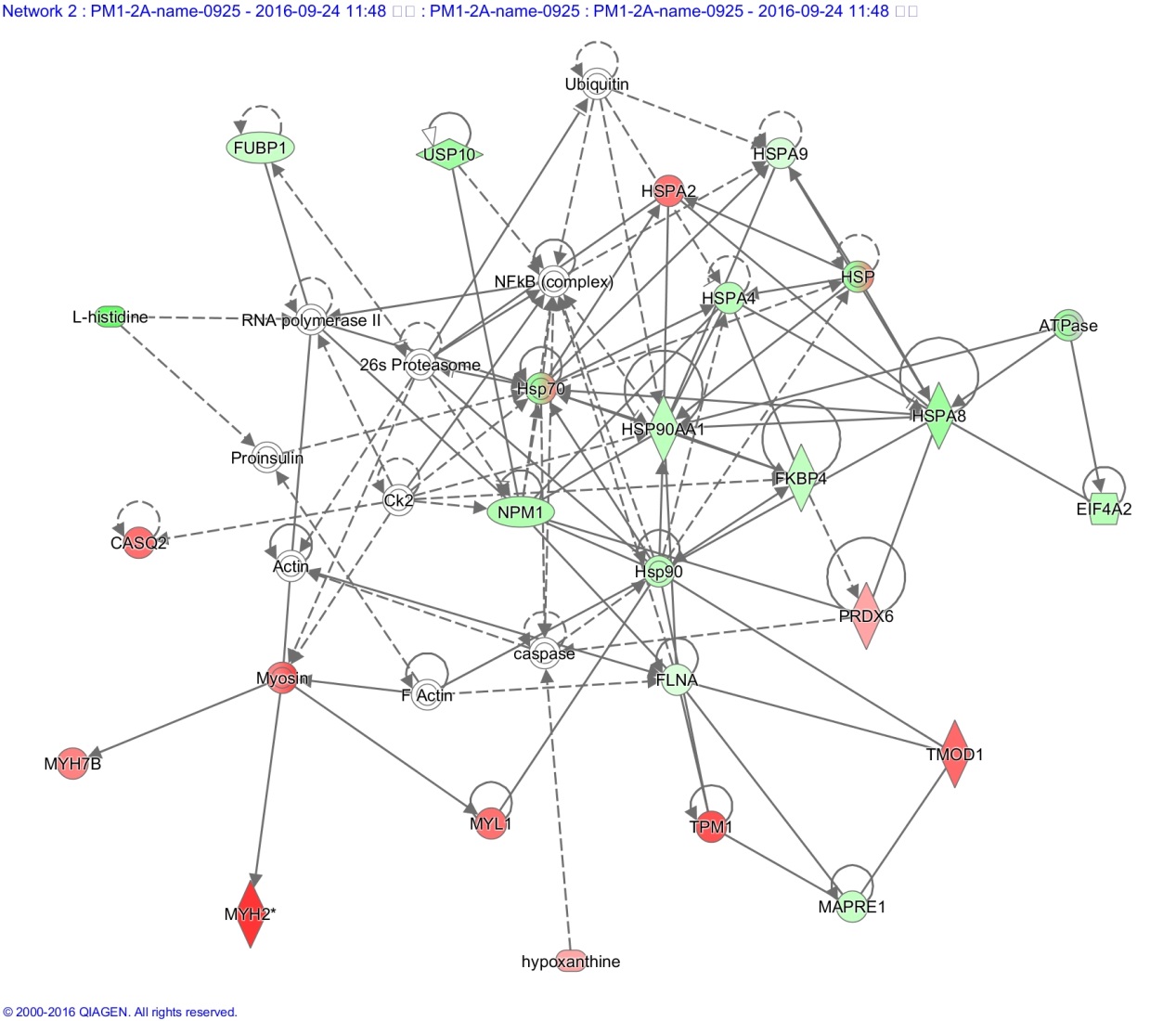


**Figure S4. Top significant network enriched by DA proteins and metabolites (from ED 12 – ED 17) related to Protein Folding and Cell Morphology in BJY chickens.** Red and green symbols show the up-regulated and down-regulated molecules found in the current study while white symbols indicate genes that are functionally associated.


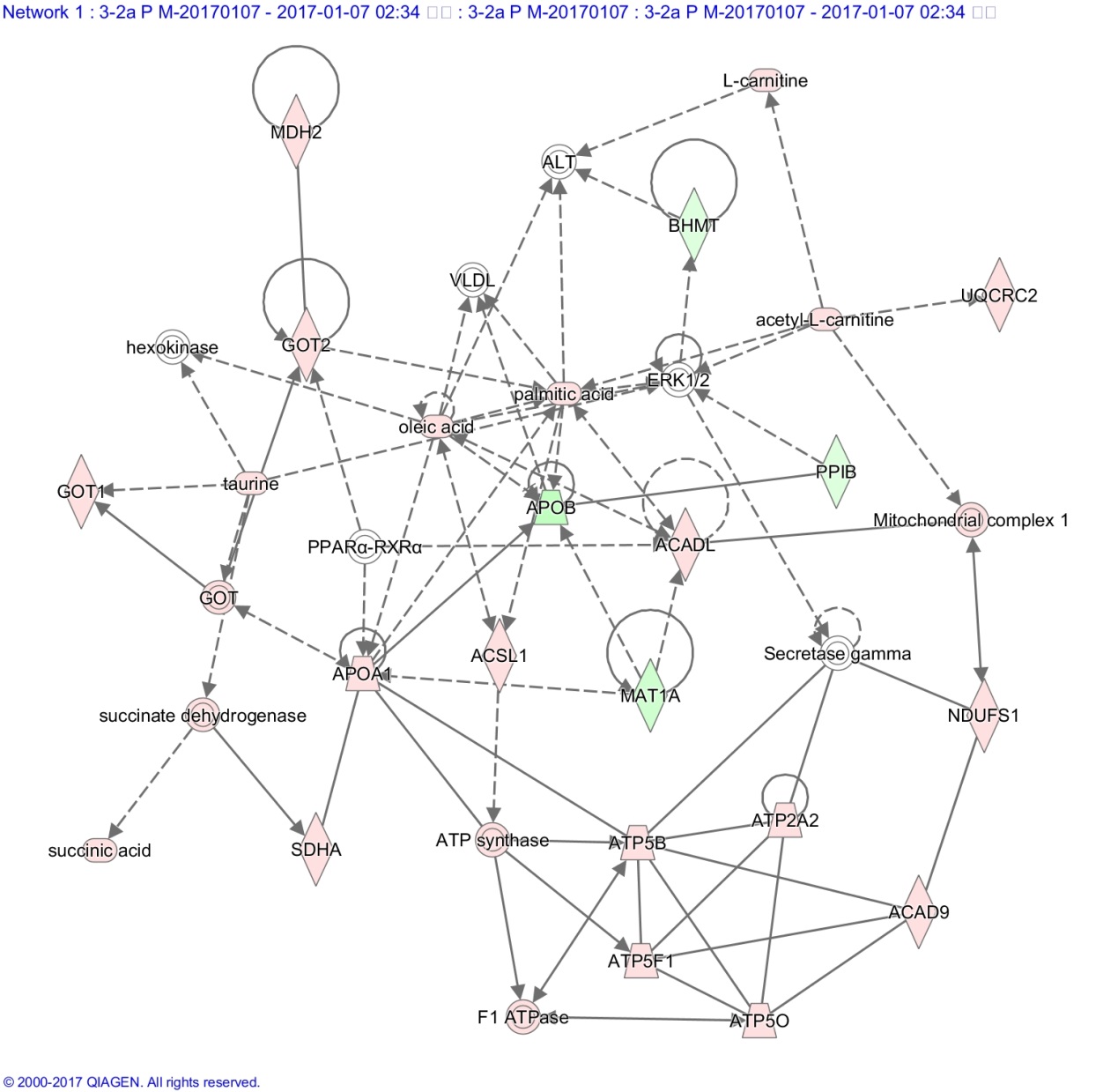


**Figure S5. Top 1 Significant network (Top 1) enriched by DA proteins and metabolites (from ED 17 – D 1) related to Lipid Metabolism in BJY chickens.** Red and green symbols show the up-regulated and down-regulated molecules found in the current study while white symbols indicate genes that are functionally associated.
